# Changes in pancreatic histology, insulin secretion and oxidative status in diabetic rats following treatment with *Ficus deltoidea* and vitexin

**DOI:** 10.1186/s12906-017-1762-8

**Published:** 2017-06-02

**Authors:** Samsulrizal Nurdiana, Yong Meng Goh, Hafandi Ahmad, Sulaiman Md Dom, Nur Syimal’ain Azmi, Noor Syaffinaz Noor Mohamad Zin, Mahdi Ebrahimi

**Affiliations:** 1 Faculty of Applied Sciences, Universiti Teknologi MARA, 40450 Shah Alam, Selangor Malaysia; 2 Department of Veterinary Preclinical Sciences, Faculty of Veterinary Medicine, Universiti Putra Malaysia (UPM), 43400 Serdang, Selangor Malaysia; 3 Medical Imaging Department, Faculty of Health Sciences, Universiti Teknologi MARA, Puncak Alam Campus, 42300 Puncak Alam, Selangor Malaysia

**Keywords:** Diabetes, *F. deltoidea*, Vitexin, Ft-Ir, Pancreas

## Abstract

**Background:**

The potential application of *Ficus deltoidea* and vitexin for the management of symptomatologies associated with diabetes mellitus (DM) has gained much attention. However, less firm evidence comes from data to augment our understanding of the role of *F. deltoidea* and vitexin in protecting pancreatic β-cells. The aim of this study was to assess histological and oxidative stress changes in the pancreas of streptozotocin (STZ)-induced diabetic rats following *F. deltoidea* extract and vitexin treatment.

**Methods:**

*F. deltoidea* and vitexin was administrated orally to six-weeks STZ-induced diabetic rats over 8 weeks period. The glucose and insulin tolerances were assessed by intraperitoneal glucose (2 g/kg) tolerance test (IPGTT) and intraperitoneal insulin (0.65 U/kg) tolerance test (IPITT), respectively. Subsequently, insulin resistance was assessed by homeostasis assessment model of insulin resistance (HOMA-IR), quantitative insulin sensitivity check index (QUICKI) and the insulin/triglyceride-derived McAuley index. The histological changes in the pancreas were then observed by hematoxylin-eosin (H&E) staining. Further, the pattern of fatty acid composition and infrared (IR) spectra of the serum and pancreas were monitored by gas chromatography (GC) method and Fourier Transform Infrared (FT-IR) spectroscopy.

**Results:**

*F. deltoidea* and vitexin increased pancreatic antioxidant enzymes and promoted islet regeneration. However, a significant increase in insulin secretion was observed only in rats treated with *F. deltoidea*. More importantly, reduction of fasting blood glucose is consistent with reduced FT-IR peaks at 1200-1000 cm^−1^.

**Conclusions:**

These results accentuate that *F. deltoidea* and vitexin could be a potential agent to attenuate pancreatic oxidative damage and advocate their therapeutic potential for treating DM.

## Background

Hyperglycaemia is the predominant cause of diabetic complications [1, 2]. Despite optimal treatment regimens, glucose fluctuation in the person with diabetes remains a major challenge [3]. Acute glucose fluctuation in turn is responsible to influence the magnitude of oxidative stress. Importantly, oxidative stress has been postulated as a possible mechanism for diabetes-associated tissue and other systemic complications [4]. It is thus justified to conclude that replenishment of insulin-producing pancreatic β-cells is crucial for treating diabetes and its complications [5], thereby overcoming the inadequacies of current treatment strategies.

It is now recognized that both major types of DM affects β-cells mass and insulin secretion [6]. This fact has prompted renewed interest in targeting the pancreatic β-cell. The appreciation of the pancreatic β-cell offer promise in improving glycaemia control as well as potentially reducing the progression of diabetic complications. In most cases a single large dose of STZ has been carried out on laboratory animals for experiments attempting to determine strategies for replenishing β-cells [7, 8].

In Malaysia, *F. deltoidea* is frequently used for multiple purposes due to the high antioxidant activities. There is a large volume of published studies describing that the methanolic extracts of *F. deltoidea* plant leaves are rich sources of polyphenolics, flavonoids, and tannins [9, 10]. Vitexin was identified as a compound marker that associated with antioxidant and antidiabetic properties of *F. deltoidea* in animal models [11]. *F. deltoidea* are known to be involved in lowering blood glucose level by enhancing the hepatic glycolytic enzymes in type 1 DM rats [12]. There is also evidence that *F. deltoidea* [13, 14] and vitexin [11, 15] inhibits in-vivo and in-vitro α-glycosidase activity. In greater detail, Farsi et al. [16] revealed that *F. deltoidea* stimulates insulin secretion and blocks hepatic glucose production by regulating the expression of hepatic GK and PPARγ genes. Taken together, these findings imply that *F. deltoidea* and vitexin mimic the glucose-lowering effect of metformin as a popular synthetic drug for DM [17, 18]. Strikingly, new data showed the possibility of metformin to chemically protect β-cells survival [19]. However, the information pertaining to the effects *F. deltoidea* and vitexin on the histological changes in the pancreas of rats are rather limited and inconclusive.

On the basis of these considerations, the present study was conducted to characterize histological and oxidative stress changes in the pancreatic of STZ-induced diabetic rats following *F. deltoidea* and vitexin treatment. In this study, it was also determined whether changes in tissue and blood serum would alter the composition of fatty acid and the pattern of FT-IR spectral.

## Methods

### Plant material and extract preparation

The leaves of *F. deltoidea* var. *deltoidea* were collected from Forest Research Institute Malaysia, Kepong, Malaysia in January 2015. The sample was then deposited at the Herbarium Unit, Universiti Kebangsaan Malaysia, Bangi and identified by Mr. Sani Miran with a voucher number Herbarium UKMB-40315. The leaves were washed thoroughly and over-dried at 37 ± 5 °C. The dried leaves were finely powdered using an electric grinder. For extraction, 100 g of powdered leaves was soaked in 1 L absolute methanol for three days at room temperature [16]. Liquid extracts were concentrated using a rotary evaporator at 40 °C and subjected to freeze drying for 48 h. The extraction yield calculated was 10.6%.The extracts were kept in tightly closed glass containers and stored at −20 °C until further use.

### Animals

The animal use and experimental protocols involved in the study were approved by the Universiti Putra Malaysia, Animal Care and Use Committee with an approval number: UPM/IACUC/AUP-R090/2014. A total of 30 male Sprague Dawley rats of four-week-old (mean body weight, 100 ± 5 g) were procured from Chenur Supplier Sdn. Bhd., Serdang, Selangor. The rats were housed at Laboratory Animal Facility and Management (LAFAM), Universiti Teknologi MARA, Puncak Alam, Selangor. The animals were acclimatized upon arrival for a week and were housed at a density of three per cage in a temperature controlled room (22 ± 1 °C and a 12 h light/dark cycle). The blood glucose levels and body weights of all animals were measured at the beginning of the study. The rats were identified with a cage card indicating project number, dose level, group, and animal number. They had access to standard rat chow (Gold Coin Holdings, Kuala Lumpur, Malaysia) and water ad libitum.

Diabetes-like hyperglycaemia was induced experimentally in rats through intraperitoneal injection of 0.5 ml STZ (Sigma-Aldrich, Deisenhofen, Germany) at a dosage of 60 mg/kg of body weight (b.w.) [20]. After a week, animals with fasting blood glucose levels >11 mmol/L were considered diabetic [21].

### Experimental design and procedure

The rats were divided into five groups of six rats per treatment group. The treatment group were normal control rats received saline (NC), diabetic control rats received saline (DC), Diabetic rats treated with 1000 mg/kg b.w. of metformin (DMET) [22], diabetic rats treated with 1000 mg/kg b.w. of *F.deltoidea* (DFD) [16], diabetic rats treated with 1 mg/kg b.w. of vitexin (DV) [11].

Metformin, methanolic extract of *F. deltoidea* and vitexin were dissolved in saline and treatments were given once daily via oral gavage for 8 weeks. Blood was sampled from the tail vein and fasting blood glucose was measured using a portable glucometer (Accu-Chek, Roche, Germany) at 1-week intervals.

At the end of the experiment, all animals were fasted overnight. Animals were then anesthetized with ketamine (80 mg/kg) and xylazine (8 mg/kg), followed by terminal exsanguination. Blood samples (10-15 ml) were collected via cardiac puncture from the rats into plain red-top tube containing no anticoagulants (BD Vacutainer®, USA). The blood samples were then centrifuged at 4000  g for 15 minutes, and serum was stored in aliquots at −80 °C. Triglycerides in serum were determined using an automatic analyser (Hitachi 911, Boehringer-Mannheim, Germany). Meanwhile, pancreas was carefully excised, rinsed in ice-cold saline and stored in 10% formalin for tissue characterization.

### Glucose and insulin tolerence measurements

The intraperitoneal glucose tolerance test (IPGTT) and intraperitoneal insulin tolerance test (IPITT) were performed at the end of 8 weeks treatment on all experimental groups. These tests were performed with minor modifications following the method described by Abdollahi [23]. The rats were fasted for 8 h prior to testing. For the IPGTT, glucose (2 g/kg) was injected intraperitoneally at time 0. Following day, the rats were intraperitoneally injected with insulin (1.5 IU/kg) for the IPITT. Blood glucose measurements from the tails were performed at 0, 30, 60 and 120 min. The blood glucose concentration versus time (minutes) was plotted and the area under curve (AUC) was calculated following the trapezoidal rule.

### Fasting serum insulin

The levels of serum insulin were determined by Enzyme Linked Immunosorbent Assay kit specific for rat insulin (Cloud-Clone Corp., Houston, USA) as described by Zhang et al. [24]. Serum was allowed to thaw and 50 μl serum was pipetted into duplicate wells followed by 50 μl of enzyme conjugate solution. This mixture was incubated for 1 h on a plate shaker (800 rpm) at 37 °C. The plate was inverted on an absorbent paper after the final wash. Afterward, 90 μl of substrate solution was pipetted and incubated at 37 °C. Finally, the reaction was stopped 15 min later by adding 50 μl of stop solution to each well. The optical density was read at 450 nm with microplate reader (Epoch Microplate Spectrophotometer, BioTek, USA). The values for the calibrators were used to plot a calibrator curve from which the values for the samples were extrapolated.

### Determination of insulin sensitivity

Three indirect indexes for the assessment of insulin sensitivity were calculated using serum insulin, glucose and triglyceride at the end of the experimental period. The HOMA index uses the formula described by Matthews et al. [25] while the quantitative insulin sensitivity check index (QUICKI) is based on the logarithmic transformation. Accordingly, McAuley’s index [26] was calculated based on the increase of triglycerides levels and insulin according to the following equations:

$$ \mathrm{HOMA}-\mathrm{IR}=\frac{\mathrm{Fasting}\ \mathrm{insulin}\ \left(\frac{\upmu \mathrm{IU}}{\mathrm{mL}}\right)\times \mathrm{fasting}\ \mathrm{glucose}\ \left(\frac{\mathrm{mmol}}{\mathrm{L}}\right)}{22.5} $$

$$ \mathrm{HOMA}-\mathrm{B}=\frac{20\times \mathrm{Fasting}\ \mathrm{insulin}\ \left(\frac{\upmu \mathrm{IU}}{\mathrm{mL}}\right)\ }{\mathrm{fasting}\ \mathrm{glucose}\ \left(\frac{\mathrm{mmol}}{\mathrm{L}}\right)-3.5} $$

$$ \mathrm{QUICKI}=\frac{\ 1}{ \log fasting\  insulin\ \left(\frac{\mu IU}{mL}\right)+ \log fasting\  glucose\ \left(\frac{mg}{dL}\right)} $$

$$ \mathrm{McAuley}\ \mathrm{index}= \exp \left[2.63-0.28 \ln fasting\  insulin\ \left(\frac{\mu IU}{mL}\right)-0.31 \ln\ triglyceride\ \left(\frac{mmol}{L}\right)\right] $$

### Histological assessment

The paraffin-embedded pancreas was sectioned at 4 μm using a semiautomated microtome (RM2155; Leica Micro-systems). The tissue sections were then mounted on glass slides using a hot plate (HI1220; Leica Microsystems). Afterward, the tissue sections were deparafinized by xylene and rehydrated by different graded ethanol dilution (100%, 90%, and 70%). The sections were stained with hematoxylin and eosin (H&E). All slides were examined using light microscopy (Motic BA410, Wetzlar, Germany) equipped with a digital camera (Moticam Pro 285A, Wetzlar, Germany) under a magnification of X200.

### Fasting amylin

The amylin level in the serum was determined by using Rat Amylin Enzyme Immunoassay Kit (RayBiotech Incorporation, USA). Standards and reagents were prepared carefully according to the procedure in the instruction manual prior the assay. One hundred microliters of anti-amylin antibody was added to each well and incubated overnight at 4 °C. The solution was then discarded and washed four times by using 1X wash buffer. A 100 μl of each standard, sample and blank (assay diluent) were added in triplicates to the wells. The wells were then covered and incubated for overnight at 4 °C. The solutions were discarded and washed four times. An amount of 100 μl HRP-streptavidin solution was added to each well and incubated for 45 min at room temperature with gentle shaking. One hundred microliters of TMB one-step substrate reagent was added to each well and allowed to incubate for 30 min at room temperature in the dark with gentle shaking. Finally, 50 μl of stop solution was added to each well and the absorbance was immediately read at 450 nm wavelength using microplate reader (Epoch 2 microplate spectrophotometer, BioTek Instruments, Inc., Vermont, USA).

### Preparation of tissue homogenates

The pancreatic tissue was homogenized in 10% (*w*/*v*) homogenizing buffer (50 mM Tris-HCl, 1.15% KCl pH 7.4) using a Teflon pestle (Glass-Col, USA) at 900 rpm. The homogenates were centrifuged at 9000 g in a refrigerated centrifuge (4 °C) for 10 min to remove nuclei and debris. The supernatant obtained was used for biochemical assays and FT-IR analysis. Protein concentration was estimated by the method of Lowry [27], using bovine serum albumin as the standard.

### Estimation of MDA levels

The levels of MDA equivalents were determined in pancreas by TBARS assay kit (Cayman, MI, USA) as describe by Hardwick et al. [28]. The absorbance was determined spectrophotometrically at wavelength of 540 nm using a spectrophotometer.

### Assessment of antioxidant enzymatic activities

Glutathione peroxidase (GPx) activity was measured using assay kit (Cayman, MI, USA). The experimental procedures were carried out according to the manufacturer’s instructions [29]. The measurement of GPx activity is based on the principle of a coupled reaction with glutathione reductase (GR). The oxidized glutathione (GSSG) formed after reduction of hydroperoxide by GPx is recycled to its reduced state by GR in the presence of NADPH. The oxidation of NADPH is accompanied by a decrease in absorbance at 340 nm. One unit of GPx was defined as the amount of enzyme that catalyzes the oxidation of 1 nmol of NADPH per minute at 25 °C.

Superoxide dismutase (SOD) activity was determined using assay kit (Cayman, MI, USA). This kit utilizes a tetrazolium salt for the detection of superoxide radicals generated by xanthine oxidase and hypoxanthine. One unit of SOD was defined as the amount of enzyme needed to produce 50% dismutation of superoxide radical.

### Fatty acid analysis

Lipids from the serum and pancreas were extracted according to the methods described by Hajjar et al. [30]. According to this method, 1 ml of serum were thawed at room temperature for 30 min and extracted using the Folch method [31] (chloroform:methanol, 2:1, *v*/v) containing butylated hydroxytoluene as antioxidant. Then, fatty acids methyl esters (FAME) were prepared using 0.66 N potassium hydroxide (KOH) in methanol and 14% methanolic boron trifluoride (BF_3_) (Sigma Chemical Co. St. Louis, Missouri, USA). The FAME were separated with an Agilent 7890A Series GC system (Agilent Technologies, Palo Alto, CA, USA) using a 30 m × 0.25 mm ID (0.20 μm film thickness) Supelco SP-2330 capillary column (Supelco, Inc., Bellefonte, PA, USA). The fatty acid proportions are expressed as percentage of total identified fatty acids. One microlitre of FAME was injected by an auto sampler into the chromatograph, equipped with a split/splitless injector and a flame ionization detector (FID) detector. The injector temperature was programmed at 250 °C and the detector temperature was 300 °C. The column temperature program initiated runs at 100 °C, for 2 min, warmed to 170 °C at 10 °C/min, held for 2 min, warmed to 200 °C at 7.5 °C/min, and then held for 20 min to facilitate optimal separation. Identification of fatty acids was carried out by comparing relative FAME peak retention times of samples to standards obtained from Sigma (St. Louis, MO, USA).

### FT-IR analysis

Infrared spectroscopic experiments were performed using a Bruker 66 V FT-IR spectrometer (Bruker Corp., MA, USA) that was equipped with a focal plane array detector. Twenty microliter of each homogenate sample was then deposited on a liquid cell (demounted cell) using a pipette according to the method reported by Demir et al. [32]. All individual FT-IR spectra were recorded over the range 4000-400 cm^−1^ at room temperature. In order to resolve the overlapped absorption components in FT-IR spectra, second derivative spectra were calculated using Savitzky–Golay algorithm. All spectra processing was performed by using OPUS 7.0 software (Bruker Optics, GmbH). The peaks of each spectrum curve were then fitted and calculated. The region enriched vibration changes were compared to existing literature database towards providing chemical information on the targeted tissues. Absorptions belonging to fatty acyl chains, proteins and carbohydrates of biological samples are basically available in the 3020-2800 cm^−1^, 1700-1500 cm^−1^ and 1200-900 cm^−1^ spectral intervals, respectively [33].

### Statistical analysis

The fasting blood glucose, IPGTT and IPITT data sets were analysed using repeated measures ANOVA. Meanwhile, one-way ANOVA analyses were done on insulin levels, insulin sensitivity indexes, oxidative stress marker, antixioxidant enzymes serum and pancreas fatty acids compositions data sets to investigate the differences among the treated groups. In both cases Duncan’s multiple comparison test was employed to elucidate significant means. Results were presented as the mean ± 1 SD. All analysis was performed at 95% confidence level.

## Results

### Fasting blood glucose

The means and standard deviations of FBG in entire groups are given in Table 1. The FBG concentrations in the DC animals were increased by 50.65% compared to initial values, confirming the validity of the diabetogenic dose of STZ in this study. It is noticeable that the treatment for 8 weeks with either metformin or *F. deltoidea* or vitexin resulted in a significant reduction in FBG. The FBG decreased by 38.03% and 47.85% following *F. deltoidea* and vitexin treatment, respectively as compared to pre-treatment values. All treated diabetic groups depict significant changes at week 6 of treatment.

**Table 1:** Effect of *F. deltoidea* and vitexin on fasting blood glucose level (mmol/L) in STZ-induced diabetic rats

Weeks	Fasting blood glucose (mmol/L) ± SD				
	NC	DC	DMET	DFD	DV
0	4.80 ± 0.30^ab,x^	20.00 ± 3.24^a,y^	29.30 ± 3.70^d,z^	27.87 ± 6.03^a,z^	30.43 ± 4.07^e,z^
1	4.70 ± 0.20^ab,x^	25.27 ± 2.86^b,y^	26.83 ± 0.49^cd,y^	27.47 ± 5.23^a,y^	27.83 ± 0.40^de,y^
2	4.83 ± 0.15^ab,x^	26.77 ± 1.27^b,y^	26.10 ± 0.35^bcd,y^	23.00 ± 2.86^ab,y^	25.07 ± 3.27^cd,y^
3	4.87 ± 0.59^ab,x^	28.27 ± 3.53^b,y^	23.87 ± 5.46^abcd,y^	21.97 ± 2.59^ab,y^	24.20 ± 3.65^cd,y^
4	5.77 ± 0.42^b,x^	29.47 ± 4.32^b,z^	24.60 ± 5.10^bcd,yz^	20.33 ± 2.30^ab,y^	25.37 ± 2.03^cd,yz^
5	5.37 ± 0.81^ab,x^	27.43 ± 2.67^b,z^	20.20 ± 2.51^abc,y^	19.60 ± 1.71^ab,y^	23.10 ± 3.65^cd,yz^
6	5.33 ± 0.35^ab,x^	30.50 ± 0.95^b,z^	17.80 ± 4.25^a,y^	18.50 ± 7.63^ab,y^	21.90 ± 2.40^bc,y^
7	4.67 ± 0.96^a,x^	26.43 ± 0.51^b,z^	17.53 ± 2.20^a,y^	16.70 ± 6.56^a,y^	17.83 ± 0.29^ab,y^
8	4.93 ± 0.21^ab,x^	30.13 ± 2.63^b,z^	19.83 ± 3.75^ab,y^	17.27 ± 4.97^a,y^	15.87 ± 2.01^a,y^

Values are mean ± 1 SD for six rats in each group. Values with different superscripts^a,b,c,d^ in a column differed significant at *p* < 0.05 due to time effects. Values with different superscripts^x,y,z^ in a row differed significant at *p* < 0.05 due to treatment effects

#### Glucose and insulin tolerance

**Fig. 1:**
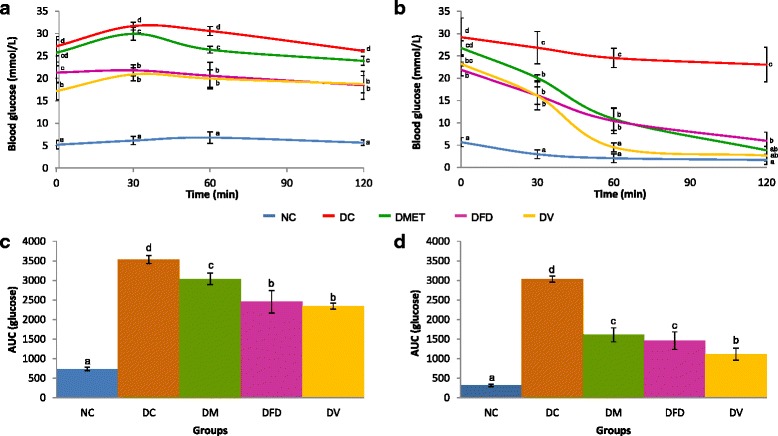
Peripheral sensitivity to glucose and insulin. Data are presented as mean ± 1 SD. **a** Glycemic values under intraperitoneal glucose tolerance test (IPGTT), **b** Blood glucose values during intraperitoneal insulin tolerance test (IPITT) in fed bats after insulin (0.5 U/kg, b.w.), **c** Area-under-the-curve (AUC) for IPGTT and (**d**) AUC for IPITT. Values with different superscripts are significantly different at *p* < 0.05

The blood glucose concentrations were measured for the period of 0-120 min after glucose and insulin infusion. The DC rats displayed impairments in glucose (Fig. 1a) and insulin responses (Fig. 1b), as characterized by increased area under the curve (AUC). Conversely, both *F. deltoidea* and vitexin treatments marginally improved whole-body blood glucose disposal in diabetic rats as indicated by lower AUCs compared to the NC group.

### Insulin sensitivity

As depicted in Table 2, serum insulin levels, HOMA-B, QUICKI and McAuley index was alleviated markedly in the DC rats compared to the NC rats. It was also found that STZ associated with a significant increase in serum triglyceride and HOMA-IR index. Notably, *F. deltoidea* increased the secretion of insulin. Adhering to three indirect indexes used to predict insulin sensitivity, both *F. deltoidea* and vitexin had a significant impact in lowering insulin resistance. Triglyceride levels also decreased in both the DFD and DV rats. Nevertheless, both treatments failed to improve the HOMA-B scores.

**Table 2:** Effect of *F. deltoidea* and vitexin on serum insulin, HOMA-IR, QUICKI and McAuley index in STZ induced diabetic rats

Parameters	NC	DC	DMET	DFD	DV
Serum insulin (μIU/mL)	4.16 ± 3.03^c^	1.58 ± 0.16^a^	1.78 ± 0.34^a^	2.41 ± 0.08^b^	1.82 ± 0.03^a^
Serum TG (mmol/L)	0.31 ± 0.02^a^	2.38 ± 0.05^c^	2.88 ± 0.52^c^	1.57 ± 0.11^b^	1.11 ± 0.18^b^
HOMA-IR	0.90 ± 0.21^a^	2.14 ± 0.03^d^	1.52 ± 0.12^bc^	1.71 ± 0.14^c^	1.36 ± 0.11^b^
HOMA-B	60.86 ± 14.33^b^	1.19 ± 0.27^a^	2.38 ± 1.20^a^	3.93 ± 0.74^a^	2.77 ± 0.40^a^
QUICKI	0.39 ± 0.01^d^	0.34 ± 0.01^a^	0.36 ± 0.01^bc^	0.35 ± 0.02^b^	0.37 ± 0.02^c^
McAuley index	13.46 ± 0.42^c^	9.34 ± 0.33^a^	8.58 ± 0.95^a^	9.43 ± 0.30^a^	11.38 ± 0.53^b^

Values are mean ± 1 SD for six rats in each group. Values with different superscripts in a row differed significantly at *p* < 0.05

### Pancreas histology

**Fig. 2:**
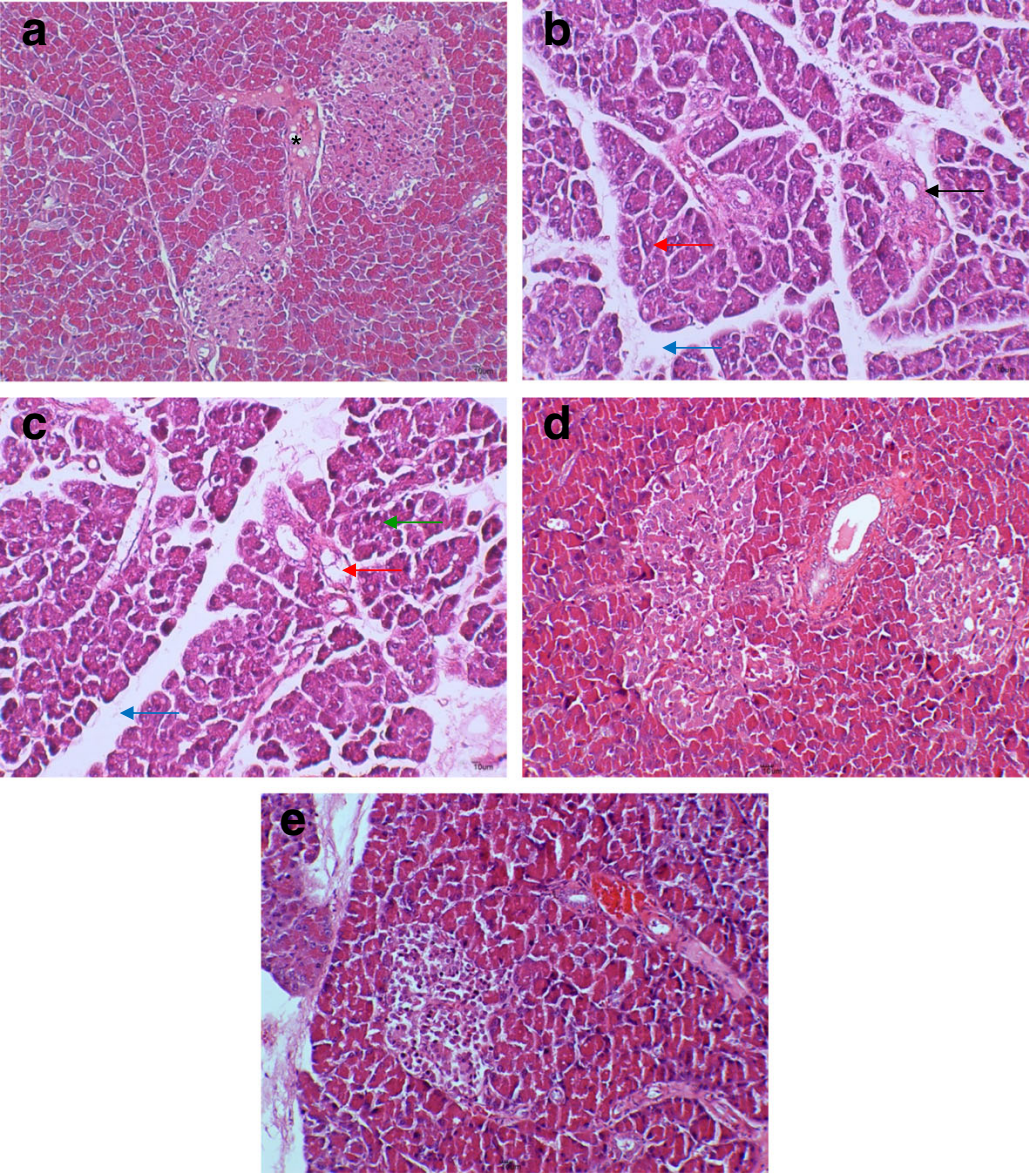
Light photomicrographs of pancreatic sections from different experimental groups. **a** Control group showing normal architecture of the pancreas. The exocrine component forms of pancreas closely packed by acinar cells and arranged into small lobules. Pancreatic lobules separated by intact intralobular and interlobular connective tissue septa. The islet cells are seen interspersed between the acinar cells. The islets appeared lightly stained than the surrounding acinar cells. **b** Diabetic rats revealed pathological changes of both exocrine and endocrine components. The acinar cells were swollen and small vacuoles were observed in almost all acinar cells. Interlobular ducts were lined with flattened epithelium [indicated by *black arrow*]. Islet β-cells are almost entirely lost in STZ-treated rats. **c** Diabetic rats treated with metformin showing distortion of the general architecture. Most exocrine acini revealed acinar damage represented by cytoplasmic vacuolation and cell atrophy [indicated by *green arrow*]. Wider interlobular [indicated by *red arrow*] and intralobular [indicated by *blue arrow*] duct were observed. **d** Diabetic rats treated with *F. deltoidea* displaying nearly normal structure of Islets of Langerhans. Atrophic change of the acinar cells was less severe and the border between exocrine and endocrine portions became more distinct. **e** Diabetic rats treated with vitexin revealed regeneration of islets. The small vacuoles in the basal area of acinar cells were also much smaller. Images are representative of three animals per experimental group (magnification ×200)

The histopathology of rat pancreas was shown in Fig. 2. Microscopic investigation of pancreas sections of NC rats showed the normal appearance of islets of Langerhans. The islets appeared lightly stained than the surrounding acinar cells. The acinar cells are formed of pyramidal cells with basal nuclei and apical acidophilic cytoplasm (Fig. 2a). However, the DC rats showed pathological changes of both exocrine and endocrine components. The acinar cells were swollen and small vacuoles were observed in almost all acinar cells. Interlobular ducts were lined with flattened epithelium. Islet β-cells are almost entirely lost in STZ-treated rats (Fig. 2b). Similar findings were obtained in the DMET rats. In fact, both the DC and DMET pancreas associated with different intensity of eosin as compared to the NC rats (Fig. 2c). On the other hand, DFD and DV groups depicted evidence of cellular regeneration among the islets of Langerhans (Fig. 2d and e). Atrophic change of the acinar cells was less severe and the border between exocrine and endocrine portions became more distinct.

### Oxidative stress marker and antioxidant enzymes

Table 3 summarizes the results for the effect of *F. deltoidea* and vitexin on pancreas lipid peroxidation and the activities of antioxidant enzymes. There was a significant decrease in the activity of SOD and GPx upon injection of STZ, reflecting the depletion of endogenous antioxidant enzymes activities in serum. Strikingly, administration of *F. deltoidea* to diabetic rats resulted in a significant increase in the SOD and GPx values relative to the DC group. Although GPx markedly increased following vitexin treatment, the SOD activity remained low throughout the studied period. Nevertheless, the DFD and DV groups associated with a significant reduction in pancreatic TBARS.

**Table 3:** Oxidative stress marker and antioxidant enzymes of various experimental groups

Groups	Oxidative stress marker	Antioxidant enzymes	
	TBARS (nmol MDA/mg protein)	GPx (U/mg protein)	SOD (mU/mg protein)
NC	1.33 ± 1.36^a^	22.38 ± 0.10^d^	10.01 ± 0.56^c^
DC	2.15 ± 0.21^b^	7.95 ± 0.76^b^	0.64 ± 0.12^a^
DMET	2.25 ± 0.39^b^	3.24 ± 0.58^a^	1.22 ± 0.06^a^
DFD	1.44 ± 0.05^a^	15.59 ± 0.21^c^	3.05 ± 0.23^b^
DV	1.03 ± 0.02^a^	15.79 ± 0.13^c^	1.35 ± 0.05^a^

Data are presented as mean ± 1 SD. Values with different superscripts in a column differed significantly at *p* < 0.05

### Amylin levels

**Fig. 3:**
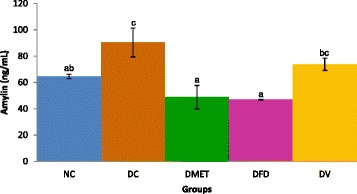
Fasting amylin. Values are presented as means ±1 SD. Bar with different alphabet notation differed significantly at *p* < 0.05

As shown in Fig. 3, serum amylin levels were significantly increased in the DC group. However, metformin and *F. deltoidea* treatments significantly reduced the levels of serum amylin.

### Serum fatty acid profiles

Table 4 lists the percentages of total fatty acids in the serum. It was demonstrated that the total serum saturated fatty acid (SFA), monounsaturated fatty acid (MUFA), and polyunsaturated fatty acids (PUFA) were roughly similar across the treatment groups. However, there were significant differences (*P* < 0.05) in the serum fatty acid profiles of DV group. The DV animals had more total n-3 PUFA in their serum as compared to the DC and NC animals. The serum docosahexaenoic acid (DHA) was clearly the highest in the DV groups.

**Table 4:** Serum fatty acid composition (percentage of total identified fatty acids) of the experimental groups^1^

Fatty acids composition (%)	Groups				
	NC	DC	DMET	DFD	DV
Saturated fatty acid (SFA)					
Myristic acid (C14:0)	2.51 ± 1.78	2.50 ± 2.31	0.68 ± 0.69	2.52 ± 1.15	2.19 ± 1.57
Palmitic acid (C16:0)	18.16 ± 3.03	21.84 ± 2.21	21.63 ± 1.34	21.49 ± 1.95	17.47 ± 4.61
Stearic acid (C18:0)	15.67 ± 2.84	10.61 ± 2.93	8.58 ± 1.02	10.03 ± 3.25	16.00 ± 8.55
total SFA	36.34 ± 1.92	34.95 ± 2.44	30.88 ± 2.61	34.03 ± 1.42	35.67 ± 11.90
Monounsaturated fatty acid (MUFA)					
Palmitoleic acid (C16:1n7)	2.04 ± 1.33	1.69 ± 1.00	1.88 ± 2.13	1.21 ± 0.78	2.13 ± 1.98
Oleic acid (C18:1n9)	13.34 ± 3.16	16.56 ± 0.96	20.31 ± 0.11	14.90 ± 5.47	14.84 ± 8.73
total MUFA	15.38 ± 2.64	18.26 ± 1.24	22.19 ± 2.04	16.11 ± 4.69	16.97 ± 7.20
n-6 PUFA					
Linoleic acid (C18:2n6)	16.61 ± 2.51	24.96 ± 2.19	30.26 ± 4.87	23.77 ± 4.64	12.92 ± 8.73
γ-Linolenic acid (C18:3n6)	2.34 ± 0.78	1.72 ± 1.51	0.00 ± 0.00	1.51 ± 1.03	2.00 ± 1.38
Arachidonic acid (C20:4n6)	14.01 ± 4.27	13.30 ± 3.89	9.39 ± 2.42	12.91 ± 2.52	14.78 ± 6.72
total n-6 PUFA	32.95 ± 2.36	39.98 ± 3.01	39.65 ± 6.85	38.19 ± 3.27	29.69 ± 15.30
n-3 PUFA					
α- Linolenic acid (C18:3n3)	3.51 ± 0.10	1.62 ± 0 .96	2.32 ± 0.87	4.77 ± 3.88	2.57 ± 1.31
Eicosapentaenoic acid (C20:5n3)	5.71 ± 1.82	0.79 ± 1.09	1.37 ± 1.26	2.36 ± 1.12	3.57 ± 4.28
Docosapentaenoic acid (C22:5n3)	3.77 ± 2.25	1.78 ± 0.580	1.23 ± 0.76	1.95 ± 0.91	5.11 ± 3.20
Docosahexaenoic acid (C22:6n3)	2.33 ± 0.83^a^	2.62 ± 1.87^a^	2.35 ± 1.87^a^	2.59 ± 0.75^a^	6.40 ± 0.60^b^
total n-3 PUFA	15.33 ± 2.97^b^	6.81 ± 0.20^a^	7.28 ± 3.26^a^	11.67 ± 2.79^ab^	17.66 ± 6.84^b^
n-6: n-3	2.20 ± 0.40^a^	5.86 ± 0.27^bc^	6.36 ± 3.03^c^	3.41 ± 0.95^ab^	1.88 ± 1.28^a^

Values are mean ± 1SD at *n* = 3. Different superscripts ^a,b,c,^ in a column differed significantly at *p* < 0.05. ^1^The data are expressed as the percentage of total identified fatty acids

### Fatty acid profiles of the pancreas

It is noticeable that the injection of STZ had insignificant effect on the total SFA and PUFA (Table 5). However, the DC group clearly had the lowest level of total MUFA. Similarly, the DV animals experienced significant decline of total pancreas MUFA at the end of the trial. Oleic acid was significantly decreased in both groups. However, the total n-3 PUFA levels in the pancreas of DV animals was more than three-fold and two-fold higher than those found in the NC and DC animals, respectively.

**Table 5:** Fatty acid composition (percentage of total identified fatty acids) of the pancreas of the experimental groups^1^

Fatty acids composition (%)	Groups				
	NC	DC	DMET	DFD	DV
Saturated fatty acid (SFA)					
Myristic acid (C14:0)	0.93 ± 0.89	0.86 ± 0.52	0.93 ± 0.51	0.82 ± 0.28	1.04 ± 0.38
Palmitic acid (C16:0)	22.97 ± 0.30	24.24 ± 5.24	24.67 ± 3.02	23.04 ± 3.12	25.52 ± 4.37
Stearic acid (C18:0)	3.38 ± 1.79^a^	11.42 ± 4.08^b^	7.64 ± 2.38^ab^	6.96 ± 0.18^ab^	11.33 ± 3.01^b^
total SFA	27.28 ± 1.82	36.52 ± 9.06	33.24 ± 4.33	30.83 ± 3.22	37.88 ± 7.33
Monounsaturated fatty acid (MUFA)					
Palmitoleic acid (C16:1n7)	2.32 ± 0.19	0.44 ± 0.21	1.54 ± 1.96	0.93 ± 0.60	1.14 ± 0.88
Oleic acid (C18:1n9)	36.83 ± 3.28^b^	22.00 ± 9.22^a^	27.35 ± 3.74^ab^	29.99 ± 2.52^ab^	21.67 ± 7.40^a^
total MUFA	39.15 ± 3.30^b^	22.44 ± 9.16^a^	28.89 ± 4.98^ab^	30.92 ± 2.63^ab^	22.81 ± 6.80^a^
n-6 PUFA					
Linoleic acid (C18:2n6)	30.34 ± 1.43	29.42 ± 5.78	28.66 ± 7.23	31.33 ± 3.31	23.44 ± 7.76
γ-Linolenic acid (C18:3n6)	0.22 ± 0.05	0.92 ± 0.50	0.63 ± 0.16	0.72 ± 0.19	1.01 ± 0.56
Arachidonic acid (C20:4n6)	1.27 ± 1.10	6.71 ± 4.63	4.69 ± 4.80	2.67 ± 1.28	8.69 ± 5.30
total n-6 PUFA	31.82 ± 1.69	37.05 ± 1.37	33.98 ± 7.17	34.73 ± 1.92	33.14 ± 2.70
n-3 PUFA					
α- Linolenic acid (C18:3n3)	1.08 ± 0.89	1.52 ± 0.06	1.54 ± 0.21	1.62 ± 0.17	2.30 ± 0.98
Eicosapentaenoic acid (C20:5n3)	0.17 ± 0.05	0.22 ± 0.14	0.67 ± 0.47	0.60 ± 0.36	0.99 ± 0.58
Docosapentaenoic acid (C22:5n3)	0.29 ± 0.09	1.49 ± 0.55	0.99 ± 0.48	0.63 ± 0.12	1.67 ± 0.88
Docosahexaenoic acid (C22:6n3)	0.20 ± 0.06	0.76 ± 0.18	0.69 ± 0.38	0.68 ± 0.32	1.22 ± 0.84
total n-3 PUFA	1.75 ± 0.91^a^	3.99 ± 0.57^ab^	3.89 ± 1.09^ab^	3.53 ± 0.83^a^	6.17 ± 2.22^b^
n-6: n-3	24.60 ± 18.81	9.18 ± 1.40	9.39 ± 4.50	10.10 ± 3.06	6.22 ± 3.87

Values are mean ± 1SD at *n* = 3. Different superscripts ^a,b,^ in a column differed significantly at *p* < 0.05. ^1^The data are expressed as the percentage of total identified fatty acids

### FT-IR spectra of the serum

**Fig. 4:**
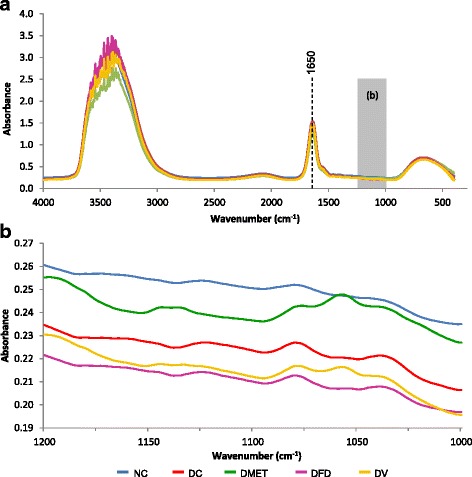
Average FT-IR absorption spectra of serum in the regions of (**a**) 4000-400 cm^−1^ (**b**) 1200-1000 cm^−1^ from different experimental groups

The FT-IR spectra changes in response to alterations in tissue and serum glucose upon *F. deltoidea* and vitexin treatment were investigated. In general, all animal groups had strong bands at 1660 cm^−1^, arising from C = O stretching vibration of the peptide group and another broad peak at 3100-3600 cm^−1^ corresponding to stretching of the hydroxyl groups (Fig. 4a). As shown in Fig. 4b, the most obvious differences between experimental groups were found in the range of 1200 and 1000 cm^−1^. This peak indicates the presence of sugar.

**Fig. 5:**
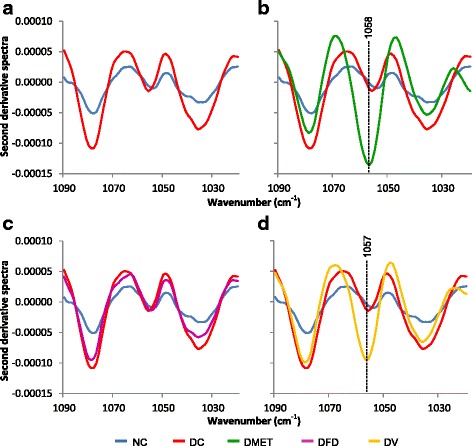
Second derivative spectra from Fig. 4 for serum in the regions of 1090-1010 cm^−1^. (**a**) Second derivative spectra between NC and DC groups. (**b**) Second derivative spectra between NC, DC and DMET groups. (**c**) Second derivative spectra between NC, DC and DFD groups. (d) Second derivative spectra between NC, DC and DV groups

The second derivative spectra below shows that the glucose peak intensities at 1080 and 1036 cm^−1^ increased in the DC rats with high blood glucose. The intensity of glucose peak was nevertheless decreased among all treated groups (Fig. 5b-d), which is consistent with the reduction of FBG concentrations (Table 1). This data indicates a possible correlation between the peak intensities at 1080 and 1036 cm^−1^ and the blood glucose concentration obtained by conventional methods. Unexpectedly, the IR spectra of serum from the DMET and DV rats elicited a greater increase in the intensity of fructose peak at 1057 cm^−1^. However, lesser intensity of fructose band was observed in the IR spectra of DFD group.

### FT-IR spectra of the pancreas

**Fig. 6:**
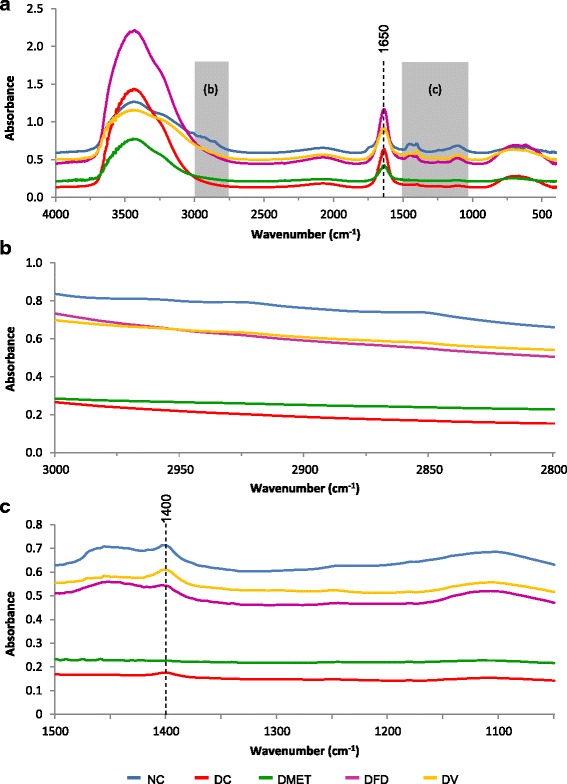
Average FTIR absorption spectra of pancreas in the regions of (**a**) 4000-400 cm^−1^ (**b**) 3000-2800 cm^−1^ and (**c**) 1500-1050 cm^−1^ from different experimental groups

The FT-IR spectra of pancreas tissues from different groups are shown in Fig. 6a. For detailed analysis of IR spectra, the region was divided into two distinct frequency ranges, namely 3000-2800 cm^−1^ (Fig. 6b) and 1500-1050 cm^−1^ (Fig. 6c). It was noticed that the IR spectra of pancreas are different at three regions which were 3000-2800 cm^−1^, 1450-1380 cm^−1^, and 1200-1000 cm^−1^. These observations imply that the structural and functional changes in pancreas are somehow related to lipid, protein and glucose specific absorption band.

**Fig. 7:**
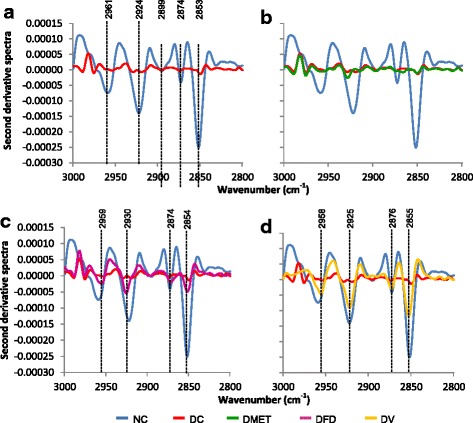
Second derivative spectra from Fig. 6 for pancreas tissue in the regions of 3000-2800 cm^−1^. (**a**) Second derivative spectra between NC and DC groups. (**b**) Second derivative spectra between NC, DC and DMET groups. (**c**) Second derivative spectra between NC, DC and DFD groups. (d) Second derivative spectra between NC, DC and DV groups

The second derivative analysis showed that the most dominant peak in the region of 3000-2800 cm^−1^ had decreased to levels far below normal in the DC and DMET groups. Interestingly, the spectra of pancreas from the DFD and DV rats are almost similar to that of the normal spectrum (Fig. 7c and d). These results suggest that lipid is involved in pancreatic regeneration.

**Fig. 8:**
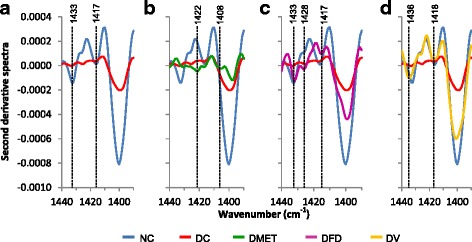
Second derivative spectra from Fig. 6 for pancreas tissue in the regions of 1440-1390 cm^−1^. (**a**) Second derivative spectra between NC and DC groups. (**b**) Second derivative spectra between NC, DC and DMET groups. (**c**) Second derivative spectra between NC, DC and DFD groups. (d) Second derivative spectra between NC, DC and DV groups

It was found that the DC animals are associated with a lower intensity of band within 1440-1390 cm^−1^ region (Fig. 8a), indicating the diminished of methyl groups for enhancing pancreatic β cell regeneration. Similar FT-IR spectra were obtained from the DMET groups as illustrated in Fig. 8b. However, second derivative spectra showed the reappearance of weak peak at 1422 and 1408 cm^−1^ in the DMET group. As seen in Fig. 8c, the spectra of DFD group displayed a clearer reappearance of the methyl band with minor shifts. Meanwhile, similar spectral characteristics seen in the NC animals illustrated in the spectra of DV group (Fig. 8d).

**Fig. 9:**
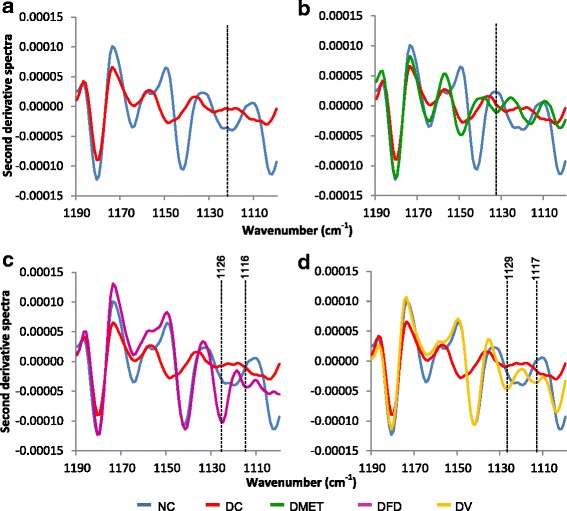
Second derivative spectra from Fig. 6 for pancreas tissue in the regions of 1190-1100 cm^−1^. (**a**) Second derivative spectra between NC and DC groups. (**b**) Second derivative spectra between NC, DC and DMET groups. (**c**) Second derivative spectra between NC, DC and DFD groups. (d) Second derivative spectra between NC, DC and DV groups

Figure 9a demonstrates that the pancreatic tissues from NC animals depict the presence of five significant peaks in the region of 1190-1100 cm^−1^. However, the intensity and frequency of the sugar band of the DC group was reduced and shifted especially between 1130 and 1106 cm^−1^. Strikingly, the spectra of DFD and DV treated groups do contain some similarities to the NC group. Both groups displayed five peaks within the region, suggesting that glucose is important elements toward the regeneration of pancreatic β-cells.

## Discussion

Profiles of IPGTT and IPITT showed that STZ induction resulted in impaired glucose and insulin tolerance. STZ induced insulin resistance on rat was then confirmed by HOMA-IR, QUICKI scores and McAuley indexes [34, 35]. In line with previous observations, the activity of pancreatic antioxidant enzymes decreased in parallel with islet cell degeneration seen in sections stained with H&E [36]. The main finding of this study was that *F. deltoidea* and vitexin are associated with regenerative effect on the islet cells. In support, the levels of FBG and serum triglyceride decreased significantly following these treatments. These findings also highlighted the changes in serum and pancreas was somehow related to fatty acid composition and FT-IR spectra.

Histological examination of the pancreas of DC rats showed a complete destruction of pancreatic islet (Fig. 2b). The acinar cells were swollen and small vacuoles were observed in almost all acinar cells. The substantial drop in HOMA-B supports the deterioration of β cell function in animal models as lower HOMA-B index reflect the failure of pancreatic β-cell function [37]. This is in agreement with studies demonstrating that a single dose of 60 mg/kg STZ is capable to induce pancreatic β-cell destruction in rats and subsequent reduction of insulin secretion [38, 39]. One particular interesting finding was that DFD and DV rats had increased the size and density of dispersed islet tissue (Fig. 2d and e). It currently accepted that targeting the pancreatic β-cell is the most promising strategies for treating diabetes [40]. Several plant extracts have been previously reported to be associated with the regeneration of pancreatic β-cells in STZ-treated diabetic rats [41–43]. More important, serum insulin levels markedly increased in the DFD rats.

Both GPx and SOD activities decreased in the pancreas of DC rats, suggesting that pancreatic oxidative stress was stimulated. Similar results have also been reported in the different animal models [44, 45]. The activities of pancreatic antioxidative enzymes (GPx and SOD) are known to be diminished in the islet cells of diabetic animals as β cells are considered to be low in antioxidant defense and susceptible to oxidative stress [46, 47]. Earlier work by Tiedge et al. [48] showed that islets contain only 2% GPX1, and 29% SOD1 activities as compared to liver. It is therefore possible that, pancreatic SOD is highly responsive to hyperglycaemia than GPx. In agreement with the findings, Zhou et al. [49] reported a significant decrease in SOD mRNA expression in pancreatic β cells of diabetic animals. These results supported the hypothesis that the acceleration of cell death could be attributed to reduced pancreatic antioxidative enzymes. Importantly, increased pancreatic antioxidant capacity was remarkable in the pancreas of DFD and DV rats.

It is interesting to note that serum amylin was slightly increased in STZ treated rats, thereby may explain partly the degeneration of the islets of Langerhans. This argument is established based on the fact that amylin is implicated in the loss of β-cells [50–52]. It has been reported that amylin induces apoptosis in pancreatic β cell by increasing the expression of c-Jun, a gene that is involved in the apoptotic pathway [53]. Cai et al. [54] later showed the elevation of amylin in acute inflammation-related pancreatic disorders. Furthermore, the results of the in vitro study demonstrated that treatment of INS-1 cells with amylin enhances cell death, inhibits cytoproliferation, and increases autophagosome formation [55]. The major findings of the current study illustrated that *F. deltoidea* inhibited the amyloid aggregation but vitexin does not. Indeed, the ability of plant extracts to inhibit the formation of amylin has been reported in several studies [56, 57]. In parallel with histological changes of the pancreas, these findings raising the possibility that amylin could be part of the trigger for β-cell regeneration [58].

Disturbances of the fatty acid composition may be critical to explain the pancreatic β-cell destruction [59]. We are proceeding to describe that endogenous production of stearic acid was increased while total MUFA (oleic acid and palmitoleic acid) was decreased in the pancreas of diabetic rats. These findings align with other studies showing that stearic acid induced endoplasmic reticulum stress of pancreatic β-cells [60]. In fact, a substantial increase in stearic acid content of pancreatic islets incubated in the presence of glucose had been previously reported [61].

The dramatic decrease in pancreas oleic acid and palmitoleic acid was also observed following STZ-induced diabetes, suggesting the progression of pancreatic β-cell death. Cnop et al. [62] demonstrated that oleic acid exert protective effects against apoptosis in the pancreas. Notably, oleic acid was more potent than palmitoleic acid against palmitic acid-induced apoptosis in pancreatic AR42J cells [63]. High concentrations of oleic acid have been pointed out to be effective in reversing the inhibitory effect in insulin production [64–66]. Nevertheless, Kudo et al. [67] provide evidence that chronic exposure to oleic acid led to the continuous excitation of β-cells, depletion of insulin storage, and impairment of glucose-stimulated insulin secretion (GSIS). This discrepancy can be justified by the fact that oleic acid increased the expression of GLUT2, which may partially contribute to the increased basal insulin secretion [68] but enhanced the levels of intracellular free Ca^2+^, which most likely accounts for the decrease of GSIS [69].

In the current study, it was shown that vitexin prevents β-cell destruction. This finding is likely due to the enrichment of endogenous n-3 fatty acid. The beneficial effects of n-3 PUFA at the pancreatic level has been previously explained by Bellenger et al. [70]. More details, Hwang et al. [71] pointed out that n-3 PUFA enrichment might partly prevent the STZ-related pancreatic islet damage by upregulating the basal activity of autophagy and improving autophagic flux disturbance. However, vitexin had no significant effect on insulin secretion.

Another important finding of the present work is that the changes in serum and tissue are supported by FT-IR peaks. These findings were consistent with previous results showing that alterations in the IR spectral signature are related to subsequent changes in tissue structure and function [72]. In particular, reduction of FBG is reflected by reduced FT-IR peaks at 1200-1000 cm^−1^. It is also observed that STZ decreased the intensity of glucose peak in the pancreas of diabetic animals, suggesting glucose deprivation within the cell. There is increasing acceptance of the idea that inadequate tissue glucose causes overproduction of ROS [73]. Indeed, slight increases in pancreatic TBARS level was also found in DC rats. Most in-vivo and in-vitro studies have demonstrated that glucose is the key for β cell replication [74–76]. However, Assmann et al. [77] showed that the effects of glucose on β-cell growth and survival are insulin dependent process.

It is also noticeable that the intensity of lipid methylene in the pancreas is markedly attenuated by diabetes. The similar effect of diabetes has been reported by Réus et al. [78]. Firmed convincing results have been published on the alterations of pancreatic structure and derangements in the lipid metabolism evoked by STZ [79]. The absence of any recognizable islets of Langerhans in response to STZ strengthens the link between pancreatic structure and methylene peak. In fact, Nolan et al. [80] showed that diabetes apparently causes lipid damage in the pancreas as it is essential for insulin secretion as well as to compensate for insulin resistance. Consistent with the earlier finding, we suggest that disappearance of methylene peak in IR spectra of diabetic pancreatic samples gave an important clue of destruction of the pancreas leading to impaired insulin secretion. The absence of methylene peaks along with glucose band in the IR spectrum of DC may further accentuate the initial interaction between role of insulin and glucose on β-cell regeneration and function. Strikingly, methylene, methyl and glucose peaks from the pancreas of both groups appeared almost similar to that of the normal spectrum, suggesting the suitability of FT-IR as a rapid and non-invasive detection method [81].

Despite the promising effects in reducing FBG and replenishment of β-cells in diabetic animals, FT-IR analysis revealed the presence of fructose peak with higher intensity in the serum of DMET and DV groups. It is important to note that fructose does not acutely raise blood glucose [82], thus, explains the reduction of FBG seen in the DM and DV groups. However, Jaiswal et al. [83] demonstrate that exposure to fructose induces cell-autonomous oxidative response through ROS production and thus impairs insulin signalling and attenuate glucose utilization. In fact, Arikawe et al. [84] revealed that high levels of fructose induce insulin resistance in rats. It has also been reported that prolonged high of fructose resulted in intracellular ATP depletion and uric acid generation. Subsequently, it may promote the development of renal injury [85]. Further studies are necessary to clarify the possibility of metformin and vitexin in developing kidney complications.

## Conclusion

In conclusion, this study demonstrated that *F. deltoidea* and vitexin had resulted in islets regeneration. These changes were accompanied by elevated pancreatic antioxidant enzymes. We essentially demonstrated that both functional and structural improvements of pancreas were also supported by composition of fatty acid and the pattern of FT-IR spectra. This raises the possibility of using *F. deltoidea* and vitexin as a valuable ancillary treatment that could add a novel layer of protection for the pancreas.

## Abbreviations

DHA: Docosahexaenoic acid
DM: Diabetes mellitus
FAME: Fatty acids methyl esters
FT-IR: Fourier Transform Infrared
GC-MS: Chromatography-mass spectrometry
GK: Glucokinase
GPx: Glutathione peroxidase
H&E: Hematoxylin-eosin
HOMA-IR: Homeostasis assessment model of insulin resistance
IPGTT: Intraperitoneal glucose tolerance test
IPITT: Intraperitoneal insulin tolerance test
IR: Infrared
KCl: Potassium chloride
MUFA: Monounsaturated fatty acid
PPARγ: Peroxisome proliferator-activated receptor γ
PUFA: Polyunsaturated fatty acids
QUICKI: Quantitative insulin sensitivity check index
SFA: Saturated fatty acid
SOD: Superoxide dismutase
STZ: Streptozotocin
TBARS: Thiobarbituric acid reactive substances
Tris- HCl: Tris hydrochloride
